# Sporadic Creutzfeldt-Jakob disease following venison exposure in a chronic wasting disease-endemic region: a zoonotic surveillance perspective

**DOI:** 10.3389/fpubh.2026.1847770

**Published:** 2026-06-24

**Authors:** Eithan Kotkowski, Jonathan Trout, Matthew Roberts, Michael Tabet, Carlayne Jackson, Sarah Horn

**Affiliations:** 1 Department of Neurology, UT Health San Antonio, San Antonio, TX, United States; 2 Research Imaging Institute, UT Health San Antonio, San Antonio, TX, United States; 3 Department of Neurology, University of Utah Health, Salt Lake City, UT, United States; 4 University of South Carolina School of Medicine Greenville, Greenville, SC, United States; 5 Division of Neurology, Prisma Health Upstate, Greenville, SC, United States; 6 Department of Psychiatry, UT Health San Antonio, San Antonio, TX, United States

**Keywords:** chronic wasting disease, Creutzfeldt–Jakob disease, prion disease, public health surveillance, rapidly progressive dementia, zoonosis

## Abstract

**Background:**

Creutzfeldt-Jakob disease (CJD) is a rare, rapidly progressive prion disease with established zoonotic transmission only in its variant form. Chronic wasting disease (CWD), a prion disease affecting cervid populations in North America, continues to expand geographically, raising public health concerns regarding potential interspecies transmission.

**Methods:**

We describe the clinical course, diagnostic evaluation, and public health investigation of a patient with confirmed sporadic CJD and long-term venison exposure from CWD-endemic regions in Louisiana. Clinical data, neuroimaging, electroencephalography, cerebrospinal fluid (CSF) biomarkers, neuropathology, and epidemiologic findings were reviewed.

**Case report:**

A 72-year-old lifelong hunter developed rapidly progressive dementia with startle myoclonus and characteristic MRI findings of cortical ribboning and basal ganglia diffusion restriction. CSF biomarkers (RT-QuIC, total tau, 14-3-3) and postmortem neuropathology confirmed sporadic CJD, MM1 subtype. During hospitalization, the patient developed posterior reversible encephalopathy syndrome (PRES), complicating radiographic interpretation. Given extensive cervid exposure and reports of similar illness among hunting peers from the same lodge, the case was reported to public health authorities. Given extensive cervid exposure and reports of similar illness among hunting peers from the same lodge, the apparent clustering of suspected prion disease prompted epidemiologic investigation and public health review.

**Conclusion:**

This case highlights diagnostic and public health challenges posed by prion disease in individuals with relevant zoonotic exposure histories. Although no evidence supporting confirmed CWD-to-human transmission was identified, the apparent clustering of suspected prion disease within a shared exposure network appropriately prompted epidemiologic investigation, structured exposure assessment, and public health review.

## 1 Introduction

Creutzfeldt–Jakob disease (CJD) is a uniformly fatal prion disease with an incidence of approximately 1-2 cases per million annually (1). Sporadic CJD accounts for the majority of cases and typically presents with rapidly progressive dementia, myoclonus, and characteristic neuroimaging findings. Molecular subtyping based on PRNP codon 129 polymorphism and protease-resistant prion protein characteristics has identified several sporadic CJD variants, with the MM1 subtype representing the most common phenotype associated with rapidly progressive cognitive decline and cortical ribboning on diffusion-weighted MRI (2). While variant CJD represents a proven zoonosis linked to bovine spongiform encephalopathy, no environmental or zoonotic source has been definitively associated with sporadic CJD (2, 3).

**Figure 1:**
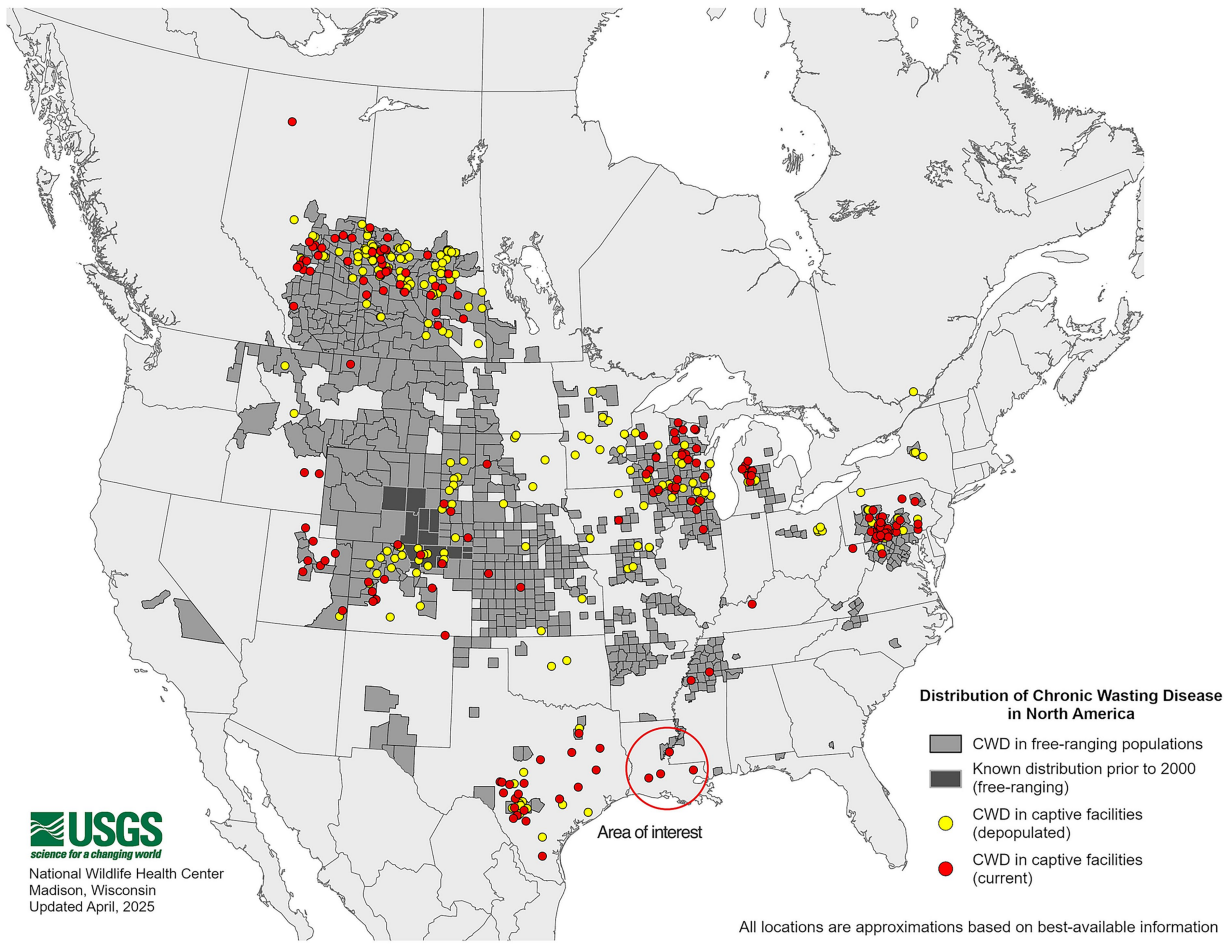
Geographic distribution of chronic wasting disease (CWD) in North America. Map adapted from the United States Geological Survey (USGS) illustrating the distribution of CWD in free-ranging and captive cervid populations (17). The circled region highlights the area of interest in Louisiana, where the patient’s hunting exposure occurred. The widespread and expanding geographic distribution of CWD underscores the relevance of zoonotic surveillance in exposed populations.

Chronic wasting disease (CWD) is an epizootic prion disease affecting deer and elk populations across North America (4) (Figure 1). Its efficient transmission among cervids, environmental persistence, and expanding geographic distribution have raised concern regarding potential human health implications as human exposure through hunting and venison consumption continues to increase. Although no confirmed human cases of CWD have been identified (5), experimental data suggest incomplete species barriers, supporting continued public health surveillance given the ongoing risk of zoonotic transmission (6, 7). Notably, experimental transmission studies in humanized animal models have demonstrated that some cervid prion strains may produce molecular and biochemical features resembling sporadic CJD MM1, complicating definitive exclusion of zoonotic overlap based solely on clinico-pathologic phenotype (7, 8).

Historically, public concern regarding zoonotic prion disease intensified following the bovine spongiform encephalopathy (BSE, or “mad cow disease”) epidemic in the United Kingdom during the 1980s and 1990s, which ultimately demonstrated confirmed transmission of animal prions to humans through the emergence of variant Creutzfeldt–Jakob disease (vCJD) (9). This event fundamentally altered scientific understanding of species barriers in prion disease and established that animal prion diseases can, under specific conditions, cross into human populations. In contrast, chronic wasting disease, first identified in captive mule deer in Colorado in 1967, has continued to expand among cervid populations throughout North America without any confirmed human cases to date (4). Nevertheless, the prolonged incubation periods characteristic of prion diseases, ongoing geographic spread of CWD, and increasing human exposure through hunting and venison consumption have sustained interest in continued surveillance and investigation of potential zoonotic transmission pathways.

Given the extreme rarity of sporadic CJD, the identification of more than one suspected case within a small, socially connected hunting lodge community represents a statistically rare event. Based on United States incidence estimates of approximately 1-2 cases per million persons per year, the probability of two independent sporadic CJD cases occurring within a small, socially connected group (e.g., 20–50 individuals) over a two-year period is on the order of 10^−8^-10^−9^, assuming independence of events (1). Under baseline United States incidence assumptions for sporadic CJD, the occurrence of multiple suspected cases within a small socially connected hunting community over a short interval would be considered epidemiologically uncommon, with estimated probabilities on the order of ~10^−8^-10^−9^ under assumptions of event independence. Accordingly, such observations may reasonably warrant further surveillance review and structured exposure assessment. Importantly, public health surveillance is not triggered by statistical rarity alone, but by the convergence of temporal proximity, shared environmental exposure, and biologic plausibility. We report a case of confirmed sporadic CJD in a lifelong hunter with extensive venison exposure from CWD-endemic regions in Louisiana, framed as a brief research report emphasizing diagnostic evaluation, exposure assessment, and epidemiologic investigation. Unlike conventional neurology case reports, this report foregrounds the downstream public health response and surveillance implications rather than diagnostic novelty alone.

## 2 Methods

### 2.1 Clinical evaluation

Clinical history, neurologic examination findings, laboratory results, EEG data, and neuroimaging were extracted from the medical record. Rapidly progressive dementia prompted evaluation for infectious, autoimmune, neoplastic, metabolic, and prion etiologies.

### 2.2 Diagnostic testing

MRI brain studies were reviewed for diffusion-weighted and FLAIR abnormalities. Continuous EEG recordings were analyzed for epileptiform activity. Cerebrospinal fluid testing included cell counts, protein, glucose, autoimmune encephalitis panels, and prion biomarkers (RT-QuIC, total tau, 14-3-3). Postmortem neuropathologic examination and *PRNP* gene testing were performed.

### 2.3 Public health investigation

Given potential zoonotic exposure, the case was reported to the Centers for Disease Control and Prevention (CDC). Epidemiologic investigation focused on hunting practices, geographic exposure, and evaluation of additional suspected cases within the same hunting lodge community. To provide epidemiologic context, a binomial approximation using United States annual sporadic CJD incidence estimates (~1.4 cases per million persons annually) was used to estimate the probability of observing ≥2 cases within a small socially connected population over a two-year interval (1, 10). These probability estimates were intended as illustrative surveillance context rather than formal inferential cluster analysis and were based on assumptions of event independence, stable background incidence, and complete case ascertainment. Interpretation is additionally limited by potential ascertainment bias and incomplete diagnostic confirmation in all suspected cases. Exposure assessment included duration of hunting activity, geographic locations, species harvested, meat processing practices, and use of personal protective measures during carcass handling, as obtained through family interview and public health review.

Family interview revealed lifelong white-tailed deer hunting since adolescence in Louisiana, as well as elk hunting exposure in Montana for approximately 15 years. The patient reportedly shared venison with hunting peers from the same lodge community and participated in field dressing practices including skinning and gutting harvested animals, although subsequent meat processing was performed commercially. Harvested animals reportedly did not undergo routine chronic wasting disease testing prior to consumption.

## 3 Case report

A 72-year-old lifelong hunter with extensive deer and elk exposure from chronic wasting disease-endemic regions in Louisiana and a medical history of hypertension, hyperlipidemia, gastroesophageal reflux disease, remote prostate cancer in remission following prostatectomy, erectile dysfunction, and recent total knee arthroplasty presented with 2 months of progressive cognitive decline, behavioral changes, gait instability, and visual disturbance consistent with rapidly progressive dementia (11, 12). Examination revealed impaired attention, disorientation, verbal perseveration, and startle-induced myoclonus. Initial EEG demonstrated lateralized periodic discharges, and MRI showed symmetric basal ganglia diffusion restriction with diffuse cortical ribboning, a characteristic imaging pattern in sporadic CJD (2).

**Figure 2:**
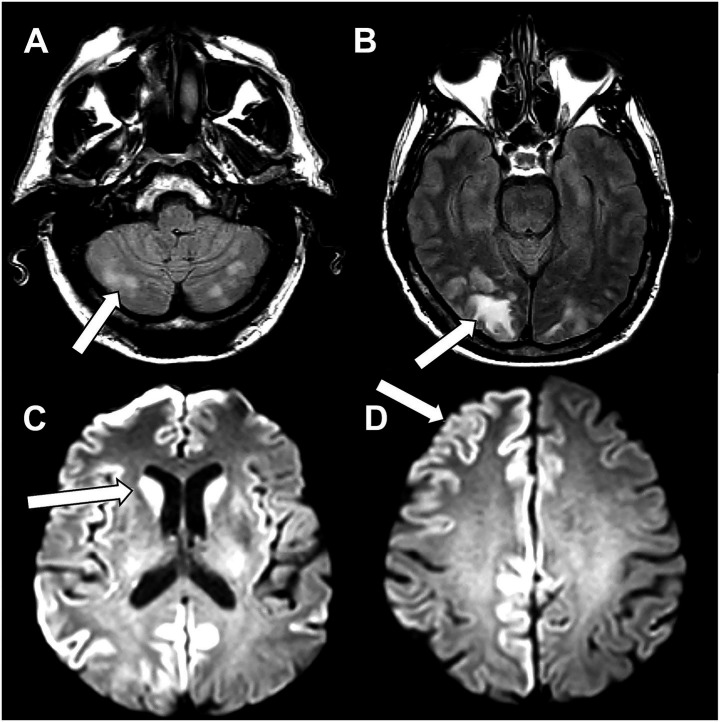
MRI findings demonstrating superimposed posterior reversible encephalopathy syndrome (PRES) in sporadic Creutzfeldt–Jakob disease (CJD). Axial FLAIR MRI sequences **(A,B)** demonstrate interval development of bilateral cerebellar and occipital cortical–subcortical T2/FLAIR hyperintensities consistent with superimposed posterior reversible encephalopathy syndrome (PRES). Diffusion-weighted imaging **(C,D)** demonstrates persistent asymmetric cortical ribboning and bilateral basal ganglia diffusion restriction characteristic of sporadic CJD, remaining largely unchanged despite interval development of PRES-related abnormalities. The coexistence of persistent prion-related diffusion abnormalities and evolving vasogenic edema complicated radiographic interpretation during the patient’s clinical deterioration.

CSF analysis revealed elevated protein without pleocytosis. Despite empiric immunotherapy, neurologic deterioration continued. Repeat EEG identified non-convulsive status epilepticus. Follow-up MRI demonstrated new bilateral occipital and cerebellar T2/FLAIR hyper-intensities consistent with posterior reversible encephalopathy syndrome (PRES), while diffusion restriction remained unchanged, a phenomenon previously reported in CJD (13) (Figure 2). The development of PRES in this patient may reflect a multifactorial process rather than a direct manifestation of prion disease alone. Potential contributing mechanisms include endothelial dysfunction related to severe neurologic injury, autonomic instability, status epilepticus, blood pressure fluctuations during intensive care management, or treatment-related factors. Experimental and neuro-pathologic studies have suggested that prion diseases may involve vascular and autonomic regulatory pathways, raising the possibility that advanced prion-related neurodegeneration could increase susceptibility to secondary cerebrovascular dysregulation (13–15). Nevertheless, given the patient’s concurrent status epilepticus and critical care course, the PRES findings are more appropriately interpreted as a likely secondary complication occurring in the setting of severe neurologic illness rather than evidence of a distinct prion-specific radiographic phenotype.

The patient died following transition to comfort-focused measures. CSF biomarkers returned positive for RT-QuIC with markedly elevated total tau and 14-3-3 protein. Neuro-pathologic examination confirmed sporadic CJD, MM1 subtype, with no pathogenic *PRNP* mutation, consistent with established molecular classifications (2). The MM1 molecular subtype represents the most common sporadic CJD phenotype and is classically associated with rapidly progressive dementia and characteristic cortical diffusion restriction on MRI, supporting classification as sporadic disease despite recognized biochemical overlap between some cervid prion strains and sCJDMM1 profiles. Key diagnostic findings are summarized in Table 1. Definitive classification in the index patient was supported by postmortem neuro-pathologic examination performed through established prion surveillance pathways. In contrast, one additional hunting lodge member reportedly demonstrated positive CSF prion biomarkers without autopsy confirmation, limiting definitive classification.

**Table 1:** Diagnostic studies supporting sporadic CJD diagnosis.

Test	Patient result	Reference range/expected normal	Interpretation
CSF RT-QuIC	Positive	Negative	Strongly supportive of prion disease
CSF total tau	>20,000 pg/mL	0–1,149 pg/mL	Markedly elevated
CSF 14-3-3 protein	Positive	Negative	Supportive of prion disease
PRNP mutation testing	Negative	No pathogenic mutation	Supports sporadic rather than genetic CJD
MRI brain DWI	Cortical ribboning + basal ganglia restriction	Absent	Characteristic for sCJD
EEG	Lateralized periodic discharges	No periodic discharges	Supportive but nonspecific

Concern for zoonotic transmission arose after the neurology team learned that other individuals from the same hunting lodge had experienced similarly rare neurologic symptoms. Public health investigation identified one additional individual from the lodge with reported positive CSF prion biomarkers but without neuro-pathologic confirmation, precluding definitive classification. This investigation allowed exclusion of alternative neurodegenerative explanations within a suspected cluster and informed appropriate classification within national prion surveillance frameworks. Geographic and temporal analyses supported classification as sporadic CJD.

## 4 Discussion

This brief research report underscores the intersection of prion diagnostics and public health surveillance in the context of expanding CWD prevalence. Although the patient’s exposure history and coincidental neurologic presentations among other lodge members raised concern for zoonotic transmission, the clinical phenotype, MM1 molecular subtype, biomarker profile, and neuro-pathologic findings overall supported classification as sporadic CJD, without identification of novel molecular or neuro-pathologic features suggestive of a distinct zoonotic prion phenotype. Nevertheless, biochemical overlap between sCJDMM1 and certain cervid prion strains complicates definitive exclusion of zoonotic disease based solely on clinico-pathologic phenotype (8).

**Figure 3:**
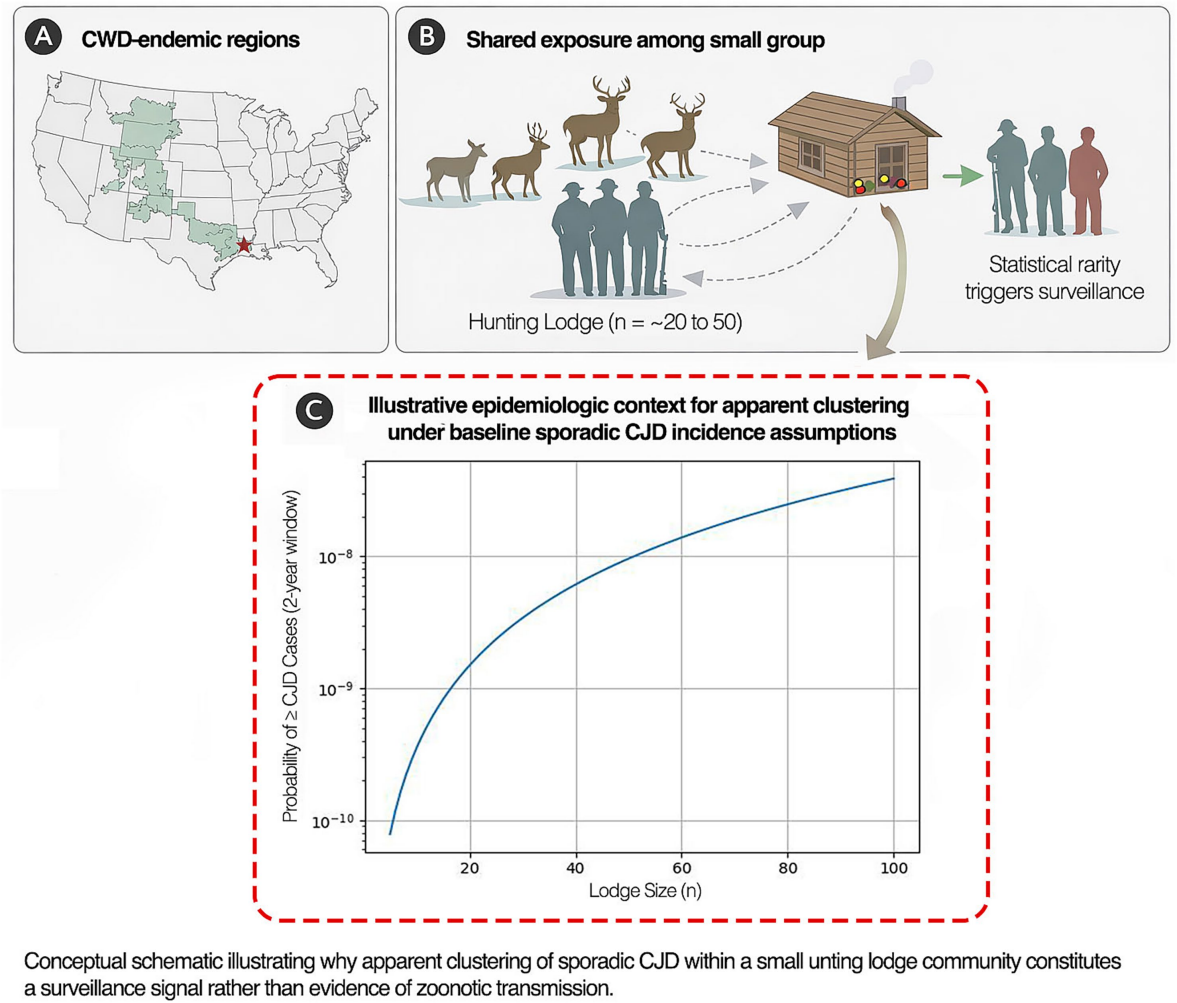
Conceptual framework for interpreting apparent clustering of sporadic CJD in the context of CWD exposure. **(A)** Geographic distribution of chronic wasting disease (CWD) highlighting endemic regions and the area of interest. **(B)** Schematic representation of shared environmental exposure within a small hunting lodge population (*n* ≈ 20–50), illustrating how social linkage and common exposure pathways may give rise to apparent clustering. **(C)** Probability of observing ≥2 sporadic Creutzfeldt–Jakob disease (CJD) cases within small populations over a two-year interval, based on United States background incidence (~1-2 cases per million per year). The logarithmic scale illustrates the low expected frequency of apparent clustering under baseline sporadic CJD incidence assumptions. Together, this framework illustrates that apparent clustering represents a surveillance signal driven by shared exposure and epidemiologic context rather than evidence of zoonotic transmission.

From an epidemiologic standpoint, the apparent clustering of suspected prion disease within a single hunting lodge warrants careful interpretation. Under baseline United States incidence rates (~1-2 cases per million per year), the probability of observing two independent cases within a small, socially connected group over a short interval (e.g., 2 years) would be considered epidemiologically uncommon under baseline sporadic CJD incidence assumptions (~10^−8^-10^−9^ assuming independence of events) (Figure 3). While the statistical probability of such an occurrence is exceedingly low, public health surveillance is not triggered by probability alone, but by the convergence of temporal proximity, shared environmental exposure, and social linkage. Importantly, these statistical estimates should not be interpreted as evidence of zoonotic transmission or causal association. Rather, they provide epidemiologic context illustrating why temporally proximate cases occurring within a socially linked exposure network may appropriately prompt public health investigation despite ultimately being classified as sporadic disease. Notably, these probability estimates assume independence and do not account for potential shared exposures, ascertainment bias, incomplete diagnostic confirmation, or additional environmental and occupational factors (e.g., including regional dietary practices, water sources, agricultural chemical exposure, and other potential confounders) that were not systematically assessed as part of this retrospective investigation. Definitive classification of human prion disease requires neuro-pathologic confirmation, and incomplete tissue availability among suspected cluster cases represents an inherent limitation of retrospective surveillance investigations. Definitive classification of human prion disease requires neuro-pathologic confirmation, and incomplete tissue availability among suspected cluster cases represents an inherent limitation of retrospective surveillance investigations. Additional environmental and occupational exposures, including regional dietary practices, water sources, agricultural chemical exposure, and other potential confounders, were not systematically assessed as part of this retrospective public health investigation and therefore cannot be excluded. Current surveillance data have not demonstrated an increased incidence of CJD in regions endemic for chronic wasting disease, and no causal link between cervid prions and human disease has been established (5, 7, 16). Accordingly, while the observed clustering appropriately prompted epidemiologic investigation, it should not be interpreted as evidence of zoonotic transmission.

This case further illustrates the value of structured zoonotic exposure assessment and timely public health notification. As CWD continues to spread among cervid populations, clinicians should remain vigilant when evaluating rapidly progressive dementia in individuals with relevant environmental exposures (6). These findings support a surveillance-based framework for evaluating potential prion disease clustering, emphasizing structured exposure assessment and epidemiologic context over statistical rarity alone (10).

## Data Availability

The original contributions presented in the study are included in the article/supplementary material, further inquiries can be directed to the corresponding author.
